# Reduced tumour growth of the human colonic cancer cell lines COLO-320 and HT-29 in vivo by dietary n-3 lipids.

**DOI:** 10.1038/bjc.1990.370

**Published:** 1990-11

**Authors:** M. Sakaguchi, S. Rowley, N. Kane, C. Imray, A. Davies, C. Jones, M. Newbold, M. R. Keighley, P. Baker, J. P. Neoptolemos

**Affiliations:** Academic Department of Surgery, Dudley Road Hospital, Birmingham, UK.

## Abstract

Seventy-five nude mice received subcutaneous inoculation with 1 X 10(7) cells of the human colonic cancer cell lines COLO-320 or HT-29. Tumour growth was assessed over 4 weeks in animals given one of three iso-caloric diets; standard diet, high saturated fat (20% coconut) diet and high n-3 fat (20% Maxepa fish oil) diet. The n-3 diet produced significant tumour growth reduction compared to the other diets for COLO-320 at 3 to 4 weeks (P less than 0.05 at least) and similarly for HT-29 at 4 weeks (P less than 0.05). Significant incorporation of n-3 fatty acids occurred in red cell membranes, adipose tissue and both neutral lipid and phospholipid fractions of tumour lipids in animals fed Maxepa (P less than 0.01 at least). This was accompanied by reduction of linoleic acid and arachidonic acid in these tissues (P less than 0.01 at least) but was most marked in the metabolically labile phospholipid fraction. There was high mitotic activity in the tumours from all the groups but there was no difference according to diet.


Br. J. Cancer (1990), 62, 742-747 © Macmillan Press Ltd., 1990

### Reduced tumour growth of the human colonic cancer cell lines COLO-320 and HT-29 *in vivo* by dietary n-3 lipids

M. Sakaguchi*, S. Rowley, N. Kane, C. Imray, A. Davies, C. Jones, M. Newbold, M.R.B. Keighley, P. Baker & J.P. Neoptolemos

*Academic Department of Surgery, Dudley Road Hospital and Clinical Research Block, University of Birmingham, Birmingham B18 7QH, UK.*

**Summary** Seventy-five nude mice received subcutaneous inoculation with $1 \times 10^7$ cells of the human colonic cancer cell lines COLO-320 or HT-29. Tumour growth was assessed over 4 weeks in animals given one of three iso-caloric diets: standard diet, high saturated fat (20% coconut) diet and high n-3 fat (20% Maxepa fish oil) diet. The n-3 diet produced significant tumour growth reduction compared to the other diets for COLO-320 at 3 to 4 weeks ($P<0.05$ at least) and similarly for HT-29 at 4 weeks ($P<0.05$). Significant incorporation of n-3 fatty acids occurred in red cell membranes, adipose tissue and both neutral lipid and phospholipid fractions of tumour lipids in animals fed Maxepa ($P<0.01$ at least). This was accompanied by reduction of linoleic acid and arachidonic acid in these tissues ($P<0.01$ at least) but was most marked in the metabolically labile phospholipid fraction. There was high mitotic activity in the tumours from all the groups but there was no difference according to diet.

Colorectal cancer is now the second commonest cause of cancer death in Westernised countries; epidemiological studies strongly point to an association with a high dietary fat intake, particularly of animal origin (Armstrong & Doll, 1975; Nicholson *et al.*, 1988). The major component of dietary fat is triacylyglcerols containing long-chain fatty acids. That high levels (~20%) of exogenous saturated fatty acids can promote colorectal cancer relative to low levels (~5%) has been shown in a number of experimental studies (Bull *et al.*, 1979; Galloway *et al.*, 1987; Nicholson *et al.*, 1990*a*; Nigro *et al.*, 1975; Reddy *et al.*, 1977).

The study of specific fats in the aetiology of colorectal cancer in humans has not been adequately determined given the crude nature of epidemiological dietary data and the difficulty in applying reliable dietary assessment techniques to large populations (Bingham, 1987). Whilst animal experiments have confirmed the tumour promoting potential of saturated fats, the role of unsaturated fatty acids is somewhat controversial. Some authors have reported that dietary fats of vegetable origin which are rich in linoleic acid (18:2, n-6) have a potent co-carcinogenic effect in experimental colon cancer not only at high dietary levels but also at low levels (Broitman *et al.*, 1977; Lochniskar *et al.*, 1985; Reddy *et al.*, 1985; Sakaguchi *et al.*, 1984; Smedley-Machlean & Nunn, 1941; Wilson & Lindsey, 1965); others have not (Dayton *et al.*, 1977; Nicholson *et al.*, 1990*b*).

Nevertheless, that differences may exist between fatty acid classes in terms of cancer promotion suggests specific structural requirements for such a process. Numerous mechanisms have been proposed, including effects on bile acids (Reddy *et al.*, 1985), direct luminal effects (Friedman *et al.*, 1989), alterations in lipid peroxidation (Begin & Das, 1986), perturbations in membrane fluidity (Brasitus *et al.*, 1985), changes in immune cytotoxicity (Schlager & Ohanian, 1980) and modulation of prostaglandin synthesis (Wickremasinghe, 1988).

Although fatty acids can be synthesised from glucose in cancer cells (Kanan *et al.*, 1980), this is reduced in the presence of an adequate exogenous supply (McGee & Spector, 1975). In cancer there is reduced re-esterification of free fatty acids in both experimental models (Ooktens *et al.*, 1986) and in patients (Legaspi *et al.*, 1987); this could result in increased peripheral utilisation of dietary fatty acids not only by the host tissues but by the cancer tissue. This therefore implies that certain exogenous fatty acids could be used as pharmacological agents and such a role has been proposed for the n-3 series of fatty acids (Begin & Das, 1986). Thus, three recent studies have shown reduced colon carcinogenesis in azoxymethane treated rodents given dietary n-3 lipids (Reddy & Maruyama, 1986; Reddy & Sugie, 1988; Minoura *et al.*, 1988).

In the present study we sought to examine the role of dietary n-3 fatty acids on the growth of two human colon cancer cell lines inoculated into nude mice; this was combined with fatty acid analysis of host and tumour tissue lipids in order to investigate underlying processes of metabolism.

### Methods

### *Animals and experimental design*

Six to eight week old BALB/c nude mice were obtained from the Department of Cancer Studies, University of Birmingham, UK, and were housed five per cage in an isolated room at 22°C. During 1 to 2 weeks acclimatisation, the mice were allowed free access to a (sterilised) standard mouse diet (Diet 10 mm (422), Pilsbury's Ltd, Birmingham, UK) with water *ad libitum*. Cohorts of animals were divided into six groups of 12 to 13 mice per group according to the type of experimental diet and tumour cell line inoculated subcutaneously (s.c.). The animals were commenced on the experimental diets the following day. Tumour measurements were undertaken at weekly intervals for the 4 subsequent weeks. They were examined daily and weighed weekly. On the fourth week (i.e. 5 weeks after cell inoculation) the animals were sacrificed and blood, subcutaneous adipose tissue and tumour were removed for lipid analysis. The tumours were weighed and divided into two for separate histological examination as well as lipid analysis. Animals with tumours which did not take following inoculation were excluded at 1 week. For humanitarian reasons, animals not sustaining health because of tumour growth were killed before 4 weeks. A further three groups of five mice each were used as controls (without tumours) to compare animal growth rates.

Preliminary studies in animals both with and without tumour inoculations were found to consume a constant 8 g of the experimental diets and standard diet for the duration of the experiment. This was therefore used during the course of the main experiment. The size of the tumour inoculation, as well as the duration of the experiment based on tumour

*Present address: Department of Surgery, Kansai Medical University, 1 Fumizono, Moriguchi, Osaka 570, Japan.
Correspondence: J.P. Neoptolemos.
Received 17 April 1990; and in revised form 18 June 1990.

growth rates, were also determined in these preliminary experiments.

*Human colon cancer cell lines*

These were obtained from the European collection of Animal Cell Cultures (Salisbury, UK). They were propagated in medium containing RPMI 1640 (90%), heat inactivated foetal bovine serum 10%, penicillin and streptomycin at 37°C in a humidified 5% $CO_2$ atmosphere.

COLO-320 HSR (ATCC CCL 220.1): is an adenocarcinoma derived from the colon and is a substrain of COLO 320 DM; it does not express carcinoembryonic antigen.

HT-29 (ATCC HTB 38): is a moderately well-differentiated adenocarcinoma derived from colon which expresses carcinoembryonic antigen.

For each animal $1 \times 10^7$ cells in 0.2 ml phosphate buffered saline (PBS) were injected into the dorsum of the chest wall subcutaneously using a 27 g needle.

*Experimental diets*

The standard diet (diet 10 mm (422) Pilsbury's Ltd, Birmingham, UK) contained the following calculated amounts: fat 5.6%, protein 20.3%, fibre 37.6%, ash 8.99%, starch 36.11% and sugar 3.04%, as well as electrolytes, trace elements and vitamins; the amount of vitamin E (dl-alpha-tocopherol acetate) was 0.07 mg $g^{-1}$. A high (20%) saturated fat diet and a high 20% n-3 fat diet was prepared by adding (W/W) coconut oil (KTC Ediblestin, Wednesbury, Birmingham, UK) and Maxepa oil (Duncan Flockhart Ltd, London, UK) to a low fat cereal based diet (Special Diet Services, Witham, Essex, UK). The base diet contained the following calculated amounts: fat 0.8%, protein 15%, fibre 5.6%, ash 4.8%, starch 54.4% and sugar 8.3% and also electrolytes, trace elements and vitamins. Both the high fat diets contained 1 mg $g^{-1}$ of vitamin E and 1 $\mu$g $g^{-1}$ of selenium. In addition, the Maxepa diet contained small amounts of dodecyl gallate and <0.01% (W/W) of cholesterol. The calculated energy value for the standard diet was 11.8 MJ $kg^{-1}$ and 12.9 MJ $kg^{-1}$ for the high fat diets. Both the high fat diets were prepared daily in order to avoid auto-oxidation of highly unsaturated fatty acids. Determination of the fatty acids in these diets was undertaken using chloroformmethanol extraction and analysis of the methyl esters using gas-liquid chromatography (see below). The fatty acid composition of these three diets is shown in Table I.

*Assessment of tumour growth*

Tumour volumes and weight were performed using minor modifications of previously described techniques (Euhus *et al.*, 1986; Fiennes, 1988). The coefficient of correlation between tumour weight and tumour volume measured by these techniques in two separate experiments was $r = 0.945$ ($n = 13$) and $r = 0.988$ ($n = 10$), both $P < 0.001$. Some divergence of the volumetric method from actual weights was noted with very large tumours due to subdermal invasion despite being situated over the rib cage.

*Analytical procedures*

At death 1.5 ml of blood was drawn into EDTA coated tubes. Red cell membranes were prepared, lipids extracted, saponified and fatty acids derivatised to the methyl esters exactly as previously described (Neoptolemos & Thomas, 1990). In all analytical procedures, samples were kept on ice and the antioxidant butylated-hydroxytoluene was used.

Adipose tissue and tumour tissue were weighed and then snap frozen and stored in liquid nitrogen. Lipids were extracted from these tissues using a technique previously described (Bligh & Dyer, 1959). In the case of tumour lipids these were separated into neutral lipid and phospholipid fractions prior to saponification. Briefly, silica Sep-Pak cartridges were primed with methanol and chloroform and loaded with 0.5 ml of the crude total lipid. Addition of 20 ml of chloroform resulted in separation of the neutral lipids followed by the phospholipid fraction with 2 ml of methanol. Complete separation was confirmed by thin layer chromatography (TLC) on silica plates using chloroform:methanol: acetic acid:distilled water (170:15:15:12) and identification with 50% sulphuric acid and charring. Subsequent treatments of these lipids was as described for the red cell membranes. With these methods the extraction of all the major fatty acids from red cells, adipose tissue and tumour tissue was 95–100% with coefficients of variation of <5%.

Fatty acid methyl esters (FAMES) were analysed by gas-liquid chromatography (GLC) using a Phillips PU 4400 chromatograph (Cambridge, UK) with a programmable temperature vapouriser (PTV), flame ionisation detector, Phillips PU 6600 data station and computer integrator and automatic injection. An open tube capillary column was used (WCOT Fused Silica, 0.22 mm × 50 M, Chrompak Ltd, Holland) either in the PTV or split mode and 1 $\mu$l sample injection. Ultra-pure gases were used and were further purified prior to entry into the chromatograph using the following filters: Oxyfilter, Moisture Filter and Charcoal Filters × 2 (Chrompack UK, Ltd, Catalogue nos. 7470–7972). The settings were as follows: $H_2$ 2.8 bar, air 2.4 bar and helium 1.5 bar (flow rate of 1.6 ml $min^{-1}$); the injection port temperature was 260°C for the split mode; PTV mode settings were 8 × 10°C initially followed by 33 × 10°C at full ramp rate (24°C $s^{-1}$) with solvent evaporation; the column temperature was 150°C initially for 2 min ramping to 2°C $min^{-1}$ to 200°C and then 5°C $min^{-1}$ to 240°C and held for 15 min; detection temperature was set at 300°C.

Identification of peaks was made by reference to retention times relative to an internal standard (margaric acid, 17:0) on samples and previous analysis using authentic standards. At least two separate runs were made per sample and their values meaned. Quantification was based on integration of clearly separated peaks.

Tumour protein content was determined using the Lowry technique (Lowry *et al.*, 1951).

*Histology*

This was undertaken on coded tissue specimens by one histopathologist (M.N.). Mitotic counts were undertaken in 50 high power fields (HPF; × 400 magnification) for each of the different groups.

**Table I** Fatty acid composition of the three experimental diets, determined as described in the methods

| Fatty acid | Experimental diets (% fatty acids) Standard diet | High saturated fat (coconut oil) | High n-3 fat (Maxepa oil) |
|---|---|---|---|
| 6:0 | <0.1 | 0.66 | <0.1 |
| 8:0 | 0.93 | 7.50 | <0.1 |
| 10:0 | 1.05 | 7.97 | <0.1 |
| 12:0 | 6.92 | 26.12 | <0.1 |
| 14:0 | 3.89 | 22.74 | 7.31 |
| 16:0 | 16.73 | 11.54 | 16.62 |
| 16:1 (n-7) | <0.1 | <0.1 | 9.70 |
| 18:0 | 16.84 | 4.60 | 3.83 |
| 18:1 (n-9) | 38.79 | 9.64 | 11.35 |
| 18:2 (n-6) | 10.57 | 3.97 | 2.46 |
| 18:3 (n-3) | <0.1 | <0.1 | 0.84 |
| 20:0 | <0.1 | <0.1 | 3.24 |
| 20:1 | <0.1 | <0.1 | 1.83 |
| 20:4 (n-6) | 2.10 | <0.1 | <0.1 |
| 20:5 (n-3) | <0.1 | <0.1 | 17.69 |
| 22:5 (n-3) | <0.1 | <0.1 | 0.60 |
| 22:6 (n-3) | <0.1 | <0.1 | 14.45 |
| Unspecified | 1.38 | 4.8 | 7.68 |
| Total saturated | 46.63 | 81.13 | 38.30 |
| Total n-3 | <0.1 | <0.1 | 35.68 |
| Total lipids % Diet (w/w) | 4.6 | 20 | 20 |

*Sources of gases and materials*

Helium CP grade (>999.999% pure), hydrogen-hydrocarbon zero grade (>99.999% pure) and air – BTCA74 (<1 v.p.m. hydrocarbons, <1 v.p.m. $CO_2$ and <0.1 v.p.m. nitric oxide) were supplied by BOC-Special Gases, UK. Silica Sep-Pak (3 ml, 500 mg) were obtained from Waters Association (Milford, MA, USA) and the TLC plates (ART 11845) from Merk (Darmstadt, Germany). Chloroform, methanol and acetic acid (all AR grade) were obtained from FSA (Bishop Meadow Road, Loughborough, UK), and ethanol from James Burrough (Witham, Essex, UK). All remaining reagents and solvents, including Lowry reagents, the internal standard and FAME authentic standards were from Sigma UK LTd (Poole, Dorset, UK).

*Statistical analysis*

Analysis of multiple groups was undertaken using the Kruskal–Wallis test; with significance thus obtained analysis between two paired groups was undertaken using the two-tailed Mann–Whitney U test (Minitab system, using the University of Birmingham IBM 3090 mainframe computer). Significance was taken as $P<0.05$, except in the case of fatty acid analysis when significance was taken as $P<0.01$ due to the large number of computations.

### Results

The weights of the animals during the course of the experiment are given in Table II and tumour sizes in Table III. The initial reduction in numbers at week 1 was due to non-tumour take. Subsequent reduction in numbers was due to poor health from tumour size. Four of eight animals with HT-29 inoculations receiving the coconut diet were sacrificed at 24 days (because of large tumours) but are included as if at 28 days. There were no significant differences between the groups in terms of change of weight. Tumour volumes and weights were significantly smaller in the Maxepa-fed animals compared to control-diet and coconut-fed animals. There were no significant differences between the tumour sizes of the last two groups.

The histology of the COLO-320 tumours revealed anaplastic high grade carcinomas with extensive necrosis. The mean ( ± s.d.) mitotic indices were: coconut group 7.1 ± 3.08 mitoses $HPF^{-1}$, Maxepa group 6.30 ± 3.37 and for the control group 6.42 ± 4.07. The histology of the HT-29 tumours revealed poorly differentiated adenocarcinomas with foci of necrosis. The mitotic indices were: coconut group 6.6 ± 4.4 mitoses $HPF^{-1}$, Maxepa group 5.7 ± 3.6 and for the control group 4.8 ± 3.0. Analysis of co-variance revealed no statistical difference. Histologically there was no distinction to be made according to dietary group.

The fatty acid content of red blood cell membranes and adipose tissue are shown in Tables IV and V respectively. Important features to note are (1) incorporation of 18:2 (n-6) in both types of tissue corresponds to dietary intakes of 18:2 (n-6) in the different groups; (2) in the Maxepa-fed animals there is a greater degree of incorporation of n-3 fatty acids in red cell membranes relative to adipose tissue.

The fatty acid content in tumour neutral lipid and phospholipid fractions are shown in Tables VI and VII respectively. In both tumour types from animals fed Maxepa, only 2:5 (n-3) and 22:6 (n-3) appeared in the neutral lipid fraction whereas 22:5 (n-3) was present in the phospholipid fraction as well. A marked reduction of 18:2 (n-6) was found in both tumour types from animals fed Maxepa compared to tumours from both the other dietary groups.

### Discussion

This study has established for the first time that it is possible to restrict the growth of two human colon cancer cell lines *in vivo* using a diet high in n-3 lipids. Significant reduction in tumour size occurred not only compared to an equivalent saturated fat diet, but also compared to an isocaloric low fat standard diet. Both the Maxepa and coconut diets were well tolerated by the experimental animals given control inoculations. The COLO-320 tumours were also well tolerated by the animals for the duration of the experiment; however, the

**Table II** Animal weights (wt) during the course of the experiment according to different cell line inoculation (or controls) and according to different dietary regimens

| | | | *Animal weights (g, mean ± s.d.)* | | | | | | | |
|---|---|---|---|---|---|---|---|---|---|---|
| | | | *Week 1* | | *Week 2* | | *Week 3* | | *Week 4* | |
| *Cell line* | *Diet* | *Number* | *(n)* | *Wt* | *(n)* | *Wt* | *(n)* | *Wt* | *(n)* | *Wt* |
| COLO-320 | Standard | 12 | (7) | 21.1±2.9 | (7) | 21.7±3.0 | (7) | 23.6±2.7 | (6) | 25.1±3.2 |
| COLO-320 | Coconut | 12 | (10) | 23.5±2.6 | (10) | 25.5±2.1 | (10) | 25.1±2.1 | (10) | 26.1±1.9 |
| COLO-320 | Mexapa | 13 | (13) | 23.1±3.2 | (13) | 24.9±3.5 | (13) | 25.2±3.1 | (13) | 25.2±3.2 |
| HT-29 | Standard | 13 | (13) | 24.5±1.5 | (13) | 23.5±1.8 | (13) | 23.8±2.0 | (11) | 24.1±2.3 |
| HT-29 | Coconut | 12 | (10) | 23.9±2.1 | (10) | 24.5±2.5 | (9) | 24.8±2.7 | (8) | 25.1±2.9 |
| HT-29 | Maxepa | 13 | (12) | 22.7±2.7 | (12) | 21.1±2.4 | (9) | 22.2±2.7 | (9) | 25.8±1.9 |
| PBS[a] | Standard | 5 | (5) | 22.6±3.1 | (5) | 23.2± 1.9 | (5) | 23.9±2.3 | (5) | 24.5±3.0 |
| PBS | Coconut | 5 | (5) | 23.1±3.6 | (5) | 21.6± 3.7 | (5) | 24.5±3.2 | (5) | 24.5±2.9 |
| PBS | Maxepa | 5 | (5) | 24.9±2.4 | (5) | 25.1 ± 2.4 | (5) | 23.4±2.4 | (5) | 24.0±1.4 |

[a]Phosphate buffered saline.

**Table III** Tumour size according to the various cell line inoculations and type of diet

| | | | *Tumour volume (ml, mean ± s.d.)* | | | | | | | | *Tumour weight (g, mean ± s.d.)* | |
|---|---|---|---|---|---|---|---|---|---|---|---|---|
| *Cell line* | *Diet* | *Number* | *(n)* | *Week 1* | *(n)* | *Week 2* | *(n)* | *Week 3* | *(n)* | *Week 4* | *(n)* | *Week 4* |
| COLO-320 | Standard | 12 | (7) | 0.025±0 | (7) | 0.074±0.049 | (7) | 0.247±0.161 | (6) | 0.597±0.167 | (6) | 0.609±0.336 |
| COLO-320 | Coconut | 12 | (10) | 0.025±0 | (10) | 0.064±0.029 | (10) | 0.143±0.050[b] | (10) | 0.605±0.384[a] | (10) | 0.599±0.341[b] |
| COLO-320 | Maxepa | 13 | (13) | 0.025±0 | (13) | 0.051±0.013 | (13) | 0.089±0.042[d] | (13) | 0.265±0.163[d] | (13) | 0.278±0.160[c] |
| HT-29 | Standard | 13 | (13) | 0.025±0 | (13) | 0.068±0.034 | (13) | 0.257±0.178 | (11) | 0.555±0.418 | (11) | 0.560±0.321 |
| HT-29 | Coconut | 12 | (10) | 0.025±0 | (10) | 0.088±0.089 | (8) | 0.329±0.235 | (8) | 0.484±0.229[a] | (8) | 0.490±0.251[a] |
| HT-29 | Maxepa | 13 | (10) | 0.025±0 | (10) | 0.051±0.035 | (9) | 0.182±0.159 | (9) | 0.268±0.161[c] | (9) | 0.265±0.163[c] |

Coconut *vs* Maxepa: [a]$P<0.05$; [b]$P<0.01$. Maxepa *vs* standard: [c]$P<0.05$; [d]$P<0.01$. Coconut *vs* standard: all not significant.

**Table IV** Fatty acid composition of red blood cell membranes according to the various cell line inoculations and type of diet

| | *COLO-320* | | | *HT-29* | | |
|---|---|---|---|---|---|---|
| *Fatty acid* | *Standard* *n = 7* | *Coconut* *n = 10* | *Maxepa* *n = 5* | *Standard* *n = 5* | *Coconut* *n = 5* | *Maxepa* *n = 5* |
| 14:0 | <0.1 | 0.8±1.0 | 0.7±0.7 | 2.1±1.4 | 0.4±0.5 | 0.1±0.1 |
| 16:0 | 30.3±0.8 | 28.8±4.0 | 25.5±5.8 | 25.4±1.9 | 25.8±3.8 | 20.1±2.5 |
| 16:1 (n-7) | <0.1 | <0.1 | 1.7±1.2[a,b] | 3.2±1.1 | 0.2±0.1 | 1.0±0.2[a] |
| 18:0 | 12.3±0.7 | 11.8±2.2 | 10.4±1.9 | 13.2±2.0 | 14.3±2.4 | 10.6±1.7 |
| 18:1 (n-9) | 10.7±0.3 | 13.8±2.3 | 8.9±1.1[a,b] | 11.1±1.0 | 16.9±2.7 | 8.7±1.1[a] |
| 18:2 (n-6) | 9.5±0.5 | 6.9±1.8 | 2.7±1.1[a,b] | 10.2±0.4 | 9.0±1.1 | 2.3±0.4[a,b] |
| 18:3 (n-6) | <0.1 | 0.7±1.3 | 0.2±0.3 | <0.1 | <0.1 | <0.1 |
| 20:3 | <0.1 | 0.8±1.3 | <0.1 | <0.1 | <0.1 | <0.1 |
| 20:4 (n-6) | 26.0±0.9 | 21.3±6.6 | 8.0±2.2[a,b] | 20.7±2.4 | 19.5±1.6 | 8.5±1.5[a,b] |
| 20:5 (n-3) | <0.1 | <0.1 | 11.8±1.2[a,b] | <0.1 | <0.1 | 12.8±0.7[a,b] |
| 22:4 (n-6) | 7.0±0.3 | 13.0±4.0 | 1.0±1.0[a,b] | 5.2±1.5 | 8.8±2.0 | <0.1[a,b] |
| 22:5 (n-3) | <0.1 | <0.1 | 3.7±1.0[a,b] | <0.1 | <0.1 | 3.7±0.9[a,b] |
| 22:6 (n-3) | <0.1 | <0.1 | 23.4±3.2[a,b] | <0.1 | <0.1 | 21.4±2.5[a,b] |

Values are % mean±s.d. Maxepa *vs* coconut: [a]$P<0.01$. Maxepa *vs* standard: [b]$P<0.01$.

**Table V** Fatty acid composition of adipose tissue according to the various cell line inoculations and type of diet

| | *COLO-320* | | | *HT-29* | | |
|---|---|---|---|---|---|---|
| *Fatty acid* | *Standard* *n = 5* | *Coconut* *n = 5* | *Maxepa* *n = 5* | *Standard* *n = 5* | *Coconut* *n = 5* | *Maxepa* *n = 5* |
| 10:0 | <0.1 | 0.8±1.7 | <0.1 | <0.1 | <0.1 | <0.1 |
| 12:0 | <0.1 | 8.6±2.2 | <0.1[a] | <0.1 | 7.2±1.3 | <0.1[a] |
| 14:0 | 2.2±4.4 | 7.7±2.5 | 3.0±1.8[a] | 4.0±2.0 | 2.0±0.5 | 2.1±0.8 |
| 16:0 | 25.0±5.4 | 19.4±2.6 | 31.2±5.0[a] | 19.5±2.0 | 18.1±2.0 | 26.8±3.3[a] |
| 16:1 (n-7) | 0.9±0.9 | 6.6±2.5 | 7.5±2.4[b] | 1.3±0.9 | 4.9±0.9 | 7.0±1.7[b] |
| 18:0 | 22.4±5.6 | 6.3±4.0 | 4.0±0.8[b] | 13.4±2.9 | 10.7±2.4 | 4.5±1.3[a,b] |
| 18:1 (n-9) | 24.4±4.4 | 28.3±7.1 | 27.9±3.5 | 31.3±3.1 | 28.1±2.3 | 20.6±3.4[a,b] |
| 18:2 (n-6) | 11.3±2.5 | 7.7±2.7 | 3.2±0.8[a,b] | 10.1±1.7 | 9.9±1.2 | 4.9±1.1[a,b] |
| 20:4 (n-6) | 13.4±3.7 | 6.2±5.9 | 1.5±0.6[b] | 15.2±2.7 | 13.3±2.3 | 3.2±0.8[a,b] |
| 20:5 (n-3) | <0.1 | <0.1 | 5.8±2.4[a,b] | <0.1 | <0.1 | 10.1±0.8[a,b] |
| 22:5 (n-3) | <0.1 | <0.1 | 1.2±0.7[a,b] | <0.1 | <0.1 | 1.4±0.4[a,b] |
| 22:6 (n-3) | <0.1 | <0.1 | 9.2±2.1[a,b] | <0.1 | <0.1 | 10.2±0.3[a,b] |

Values are % mean±s.d. Maxepa *vs* coconut: [a]$P<0.01$. Maxepa *vs* standard: [b]$P<0.01$.

**Table VI** Fatty acid composition of the neutral lipid fraction from tumours in the different dietary groups

| | *COLO-320* | | | *HT-29* | | |
|---|---|---|---|---|---|---|
| *Fatty acid* | *Standard* *n = 5* | *Coconut* *n = 6* | *Maxepa* *n = 6* | *Standard* *n = 5* | *Coconut* *n = 5* | *Maxepa* *n = 5* |
| 12:0 | <0.1 | 0.54±0.43 | <0.1 | <0.1 | 0.25±0.11 | <0.1 |
| 14:0 | 0.04±0.05 | 0.23±0.23 | <0.1[a] | 0.08±0.3 | 0.30±0.17 | 0.06±0.07 |
| 16:0 | 1.72±0.17 | 1.75±0.59 | 1.96±0.24 | 0.97±0.14 | 1.23±0.13 | 1.15±0.07 |
| 16:1 (n-7) | 0.35±0.25 | 0.76±0.44 | 0.50±0.22 | 0.22±0.15 | 0.45±0.11 | 0.35±0.09 |
| 18:0 | 0.41±0.16 | 0.62±0.38 | 0.63±0.18 | 0.21±0.12 | 0.40±0.05 | 0.35±0.17 |
| 18:1 (n-9) | 2.73±0.17 | 2.20±0.97 | 2.29±0.60 | 2.01±0.12 | 1.79±0.16 | 1.26±0.26 |
| 18:2 (n-6) | 1.43±0.35 | 0.60±0.23 | 0.38±0.20[b] | 1.35±0.27 | 0.78±0.21 | 0.25±0.11[b] |
| 20:4 (n-6) | 0.29±0.23 | 0.20±0.07 | 0.15±0.06 | 1.15±0.19 | 0.61±0.25 | 0.15±0.07[a,b] |
| 20:5 (n-3) | <0.1 | <0.1 | 0.41±0.10[a,b] | <0.1 | <0.1 | 0.50±0.10[a,b] |
| 22:6 (n-3) | <0.1 | <0.1 | 0.94±0.22[a,b] | <0.1 | <0.1 | 0.92±0.24[a,b] |

Values are nmol fatty acid per mg tumour protein; mean±s.d. Maxepa *vs* coconut: [a]$P<0.01$. Maxepa *vs* standard: [b]$P<0.01$.

growth rate of HT-29 tumours was quite rapid between week 2 and week 4 necessitating early death in 11 out of 35 animals. In our experimental design we chose only to include animals in whom the inoculated cells became firmly established at 1 week. Although this occurred in only seven of 12 animals in the group inoculated with COLO-320 cells receiving a control diet, we consider this to have been a chance event. In order to improve the possibility of establishing growth, we selected a relatively high number of cells to be inoculated ($1 \times 10^7$). An alternative might be to combine a lower cell number inoculation containing stromal cells (Pritchard *et al.*, 1989); in this situation, however, it may be difficult to differentiate stromal cell growth from tumour cell growth. Even so, failure to establish growth still occurs and emphasises the value of using weekly assessment of tumour growth as undertaken in the present study.

Although the three different animal diets were well matched, there were small but potentially important differences between them. Both the high fat diets contained more vitamin E than the standard diet. The requirement for vitamin E (and other anti-oxidants) is dependent upon the dietary level of polyunsaturated fatty acids. A ratio of at least 0.6 (mg of vitamin E per g of polyunsaturated fatty acids) is required to prevent the development of vitamin E deficiency (Harris & Embree, 1963). On the other hand the presence of vitamin E which is surplus to this requirement may influence tumour growth. In *in vitro* systems, vitamin E inhibits breast tumour cell cytotoxicity induced by polyunsaturated fatty acids

Table VII Fatty acid composition of the phospholipid fraction from tumours in the different dietary groups

| | COLO-320 | | | HT-29 | | |
|---|---|---|---|---|---|---|
| Fatty acid | Standard n = 5 | Coconut n = 6 | Maxepa n = 6 | Standard n = 5 | Coconut n = 5 | Maxepa n = 5 |
| 14:0 | <0.01 | 0.24±0.13 | 0.2±0.1 | <0.01 | 0.08±0.08 | 0.02±0.01 |
| 16:0 | 0.69±0.37 | 0.64±0.17 | 0.6±0.2 | 0.57±0.19 | 0.48±0.03 | 0.38±0.07 |
| 16:1 (n-7) | <0.01 | 0.33±0.23 | 0.16±0.04 | 0.20±0.05 | 0.21±0.07 | 0.14±0.03 |
| 18:0 | 0.77±0.29 | 0.81±0.33 | 0.65±0.07 | 0.98±0.09 | 0.89±0.22 | 0.50±0.24 |
| 18:1 (n-9) | 1.98±0.50 | 1.05±0.64 | 0.86±0.20 | 1.38±0.16 | 1.17±0.27 | 1.10±0.35 |
| 18:2 (n-6) | 0.92±0.23 | 0.50±0.10 | 0.18±0.04[a,b] | 0.56±0.13 | 0.74±0.09 | 0.18±0.11[a,b] |
| 20:4 (n-6) | 0.30±0.04 | 0.57±0.14 | 0.29±0.12[a] | 0.22±0.03 | 0.91±0.16 | 0.34±0.20[a] |
| 20:5 (n-3) | <0.01 | <0.01 | 0.42±0.03[a,b] | <0.01 | <0.01 | 0.74±0.29[a,b] |
| 22:5 (n-3) | <0.01 | <0.01 | 0.37±0.18[a,b] | <0.01 | <0.01 | 0.01±0.01[a,b] |
| 22:6 (n-3) | <0.01 | <0.01 | 0.51±0.13[a,b] | <0.01 | <0.01 | 0.77±0.25[a,b] |

Values are nmol fatty acid per mg tumour protein; mean ± s.d. Maxepa *vs* coconut: [a]$P < 0.01$. Maxepa *vs* standard: [b]$P < 0.01$.

(Begin, 1987), while *in vitro* it inhibits chemically induced breast carcinogenesis (Horvarth & Ip, 1983). Nevertheless, using experimental systems involving breast cancer as a benchmark the levels of vitamin E used in all three diets were unlikely to influence tumour growth (Begin, 1987; Horvarth & Ip, 1983).

Another difference in the diets was the presence of a small amount of cholesterol in the Maxepa diet. A dietary level of 1% cholesterol may *promote* chemically-induced colorectal tumours (Broitman *et al.*, 1977; Hiramatsu *et al.*, 1983) or even less when compared to an elemental diet (Cruse *et al.*, 1984). In the present study, however, the Maxepa diet *reduced* tumour growth compared to the other two diets.

Ingested fatty acids were utilised for energy production by beta-oxidation, storage in adipose tissue and incorporation into lipid membranes. As anticipated, marked qualitative differences were found in the distribution of fatty acids in adipose tissue and red cell membranes, which were related to the three different types of dietary intake. These effects were less marked in quantitative terms in the adipose tissue which tends to store fatty acids as derived from the diet. Subsequent utilisation will be dependent upon the requirements for (1) energy and (2) incorporation into membranes which will necessitate further metabolism in order to maintain membrane fluidity by balancing the proportion of saturated to unsaturated fatty acids (Popp-Snijders *et al.*, 1986). The latter will be dependent on the main substrate polyene fatty acids derived from the diet, i.e. 18:2, n-6 or 18:3, n-3 and the preference of delta-6-desaturase for these substrates: 18:3, n-3 > 18:2, n-6, > 18:1, n-9, > 16:1, n-7 (Jeffcoat & James, 1984). As illustrated in Tables IV and V, such processes are taking place in our experimental model with regards to both cancer cell lines.

Experimental work by Kitada *et al.* (1981) has indicated that lipid mobilisation in tumour-bearing mice is largely utilised for membrane synthesis by tumours rather than energy utilisation by beta-oxidation which occurs in normal mice. The major effects shown in our experimental model are again consistent with this, although the extent to which the fatty acids are derived directly from the diet or via temporary storage in the labile fatty acid pool of adipose tissue is not quantifiable (Tables VI and VII). The most marked differences were observed in the phospholipid fraction which is the fraction most metabolically labile. In the Maxepa-fed animals both tumour types incorporated large amounts of the main n-3 fatty acids at the expense of 18:2, n-6. Whether this is simply a reflection of substrate availability or a preference for re-esterification of n-3 fatty acids is not certain. In general, the profile of tumour FAMES was more similar to the changes observed in adipose tissue compared to that occurring in the red cell membranes. What is certain is that alteration of dietary fatty acid intake will result in dramatic tumour lipid alterations. These effects may be amplified by tumour-derived lipolytic factors (Kitada *et al.*, 1981; Beck & Tisdale, 1987) which will tend to release fatty acids from the labile fatty acid pools of adipose tissue which have been recently deposited there from the diet.

Reduction in experimental colon-cancer has recently been demonstrated in rodent models receiving azoxymethane using either fish-oil supplements (Reddy & Maruyam, 1986; Reddy & Sugie, 1988) or eicosapentaenoic acid (20:5, n-3) as the ethyl ester (Minoura *et al.*, 1988). Changes in tumour lipid composition were observed similar to those reported in the current model (Reddy & Sugie, 1988; Minoura *et al.*, 1988). A previous transplanted tumour model did not demonstrate any tumour growth reduction despite apparently similar alterations in tumour lipids in animals fed fish oil (Fady *et al.*, 1988). There may be several reasons for this: (1) an unusual model has been used, i.e. the rat DHD PROb colon cell line transplated into BDIX synergenic rats; (2) the n-3 diet contained only 9% fish oil which contained only 18% n-3 fatty acids; (3) only 6.8% of the fish oil diet contained 20:5, n-3; (4) tumour lipid incorporation consisted of 7.8% 20:5, n-3 and 17.3% 22:6, n-3. This compares with a total of 30.3% n-3 fatty acids in phospholipid fraction of COLO-320 tumours fed Maxepa and 36.4% similarly in the HT-29 tumours; moreover, the 20:5, n-3 content was 9.9% and 17.8% respectively. This would suggest that in the present study almost complete saturation by n-3 fatty acids of the sn-2 position of tumour phospholipids had occurred. It would appear that both a high n-3 dietary content is required (20%), and that a high proportion of this should be 20:5, n-3 for effective tumour growth suppression.

Mitotic index analysis of both tumours surprisingly did not reveal any significant changes by diet. The mitotic activity was very high in both tumour types corresponding to the generally high growth rates. This would imply that the effect of n-3 fatty acids is to contribute to tumour cell destruction rather than to impede cellular division. The mechanism of action of fish oil remains to be elucidated. Possibilities include inhibition of cyclo-oxygenase systems (Culp *et al.*, 1979; Corey *et al.*, 1983) and increased lipid peroxidation (Begin & Das, 1986; Begin & Ells, 1987; Begin *et al.*, 1986, 1988; Cheeseman *et al.*, 1986). Moreover, in investigating these mechanisms it will be necessary to determine whether the effects are due to a specific n-3 fatty acid and conversely whether similar effects are obtained with n-6 fatty acids.

We are indebted to the Cancer Research Campaign, Dudley Road Hospital and Duncan Flockhart (Glaxo) for financial support. Mr A. Davis, Mr C. Imray, Mr J.P. Neoptolemos and Dr P. Baker are supported by the Cancer Research Campaign. We are grateful to Mrs Dilys Thomas and the Medical Illustration Department, Dudley Road Hospital for preparation of figures and the manuscript.

### References

ARMSTRONG, B. & DOLL, R. (1975). Environmental factors and cancer incidence and mortality in different countries, with special reference to dietary practices. *Int. J. Cancer*, **15**, 617.

BECK, S.A. & TISDALE, M.J. (1987). Production of lipolytic and proteolytic fators by a murine tumor-producing cachexia in the host. *Cancer Res*, **47**, 5919.

BEGIN, M.E. (1987). Effects of polyunsaturated fatty acids and their oxidation products on cell survival. *Chem. Phys. Lipids*, **45**, 269.

BEGIN, M.E. & DAS, U.N. (1986). Selected fatty acids as possible immediates for selective cytotoxic activity of anticancer agents involving oxygen radicals. *Anticancer Res.*, **6**, 291.

BEGIN, M.E. & ELLS, G. (1987). Effects of C18 fatty acids on breast carcinoma cells in culture. *Anticancer Res.*, **7**, 215.

BEGIN, M.E., ELLS, G., DAS, U.N. & HORROBIN, D.F.D. (1986). Differential killing of human carcinoma cells supplemented with n-3 and n-6 polyunsaturated fatty acids. *J. Natl Cancer Inst.*, **77**, 1053.

BEGIN, M.E., ELLS, G. & HORROBIN, D.F.D. (1988). Polyunsaturated fatty acid-induced cytotoxicity against tumor cells and its relationship to lipid peroxidation. *J. Natl Cancer Inst.*, **80**, 188.

BINGHAM, S.A. (1987). The dietary assessment of individuals: methods, accuracy, new techniques and recommendations. *Nutr. Abst. Rev.*, **57**, 705.

BLIGH, E.C. & DYER, W.J. (1959). A rapid method of total lipid extraction and purification. *Can. J. Biochem. Physiol.*, **37**, 111.

BRASITUS, T.A., DAVIDSON, N.D. & SCHACTER, D. (1985). Variations in dietary triacylglycerol saturation alter the lipid composition and fluidity of rat intestinal plasma membranes. *Biochim. Biophys. Acta.*, **812**, 460.

BROITMAN, S.A., VITALE, J.J., VAVROUSER-JAKUBA, E. & GOTTLIEB, L.S. (1977). Polyunsaturated fat, cholesterol and large bowel tumorigenesis. *Cancer*, **40**, 2455.

BULL, A.W., SOULLIER, B.K., WILSON, P.S., HAYDEN, M.T. & NIGRO, N.D. (1979). Promotion of azoxymethane-induced intestinal cancer by high-fat diet in rats. *Cancer Res.*, **39**, 4956.

CHEESEMAN, K., COLLINS, M., PROUDFOOT, K. & 4 others (1986). Studies on lipid peroxidate in normal and tumour tissues. *Biochem. J.*, **235**, 507.

COREY, E.J., SHIH, C. & LASHMAN, J.R. (1983). Docosahexaenic acid is a strong inhibitor of prostaglandin but not leukotriene biosynthesis. *Proc. Natl Acad. Sci. USA*, **80**, 3581.

CRUSE, J.P., LEWIN, M.R. & CLARK, C.G. (1984). An investigation into the mechanism of co-carcinogenesis of dietary cholesterol during the induction of colon cancer in rats by 1,2-dimethylhydrazine. *Clin. Oncol.*, **10**, 213.

CULP, B.R., TITUS, B.G. & LANDS, W.E.M. (1979). Inhibition of prostaglandin biosynthesis by eicosopentaenoic acid. *Prostaglandins Med.*, **3**, 269.

DAYTON, S., HASHIMOTO, S. & WOLLMAN, J. (1977). Effect of high-oleic and high linoleic safflower oils on mammary tumors induced by rats by 7,12-dimethylbenz(a)anthracene. *J. Nutr.*, **107**, 1353.

EUHUS, D.M., HUDD, C., LAREGINA, M.C. & JOHNSON, F.E. (1986). Tumour measurement in the nude mouse. *J. Surg. Oncol.*, **31**, 229.

FADY, C., REISSER, D., LAGADEC, P., PELLETIER OLSON, N.D. & JEANIN, J.F. (1988). *In vivo* and *in vitro* effects of fish-containing diets on colon tumour cells in rats. *Anticancer Res.*, **8**, 225.

FIENNES, A.G.T.W. (1988). Growth rate of human tumour xenografts measured in nude mice by *in vivo* cast modelling. *Br. J. Surg.*, **75**, 23.

FRIEDMAN, E., ISAKSSON, P., RAFTER, J., MARIAN, B., WINAWER, S. & NEWMARK, H. (1989). Fecal diglycerides as selective endogenous mitogens for premalignant and malignant human colonic epithelial cells. *Cancer Res.*, **49**, 544.

GALLOWAY, D.J., JARRETT, F., BOYLE, P. & 4 others (1987). Morphological and cell kinetic effects of dietary manipulation during colorectal carcinogenesis. *Gut*, **28**, 754.

HARRIS, P.L. & EMBREE, N.D. (1963). Quantitative consideration of the effect of polyunsaturated fatty acid content of the diet upon the requirements for vitamin E. *Am. J. Clin. Nutr.*, **13**, 385.

HIRAMATSU, Y., TAKADA, H., YAMAMURA, M., HIOKI, K., SAITO, K. & YAMAMOTO, M. (1983). Effect of dietary cholesterol on azoxymethane-induced colon carcinogenesis in rats. *Carcinogenesis*, **4**, 553.

HORVARTH, P.M. & IP, C. (1983). Synergistic effect of vitamin E and selenium in the chemoprevention of mammary carcinogenesis in rats. *Cancer Res.*, **43**, 5535.

JEFFCOAT, R. & JAMES, A.T. (1984). The regulation of desaturation and elongation of fatty acids in mammals. In *Fatty Acid Metabolism and Its Regulation*, Numar, S. (ed.) p. 85. Elsevier Science Publishers: Amsterdam.

KANAN, R., LYON, I. & BAKER, N. (1980). Dietary control of lipogenesis *in vivo* in host tissues and tumours of mice bearing Enrlich ascites carcinoma. *Cancer Res.*, **40**, 4606.

KITADA, S., HOYS, E.F. & MEAD, J.F. (1981). Characterization of a lipid mobilizing factor from tumours. *Prog. Lipid Res.*, **20**, 823.

LEGASPI, A., JEEVANANDANM, M., STARNES, H. & BRENNAN, M.F. (1987). Whole lipid and energy metabolism in the cancer patients. *Metabolism*, **36**, 958.

LOCKNISKAR, M., NAUSS, K.M., KAUFFMAN, P. & NEWBEANE, P.M. (1985). Interaction of dietary fat and route of carcinogen administration of 1,2-di-methylhydrazine-induced colon tumorigenesis in rats. *Carcinogenesis*, **6**, 349.

LOWRY, D.H., ROSEBROUGH, N.J., FARR, A.L. & RANDALL, R.J. (1951). Protein measurement with the Folin phenol reagent. *J. Biol. Chem.*, **193**, 265.

MCGEE, R. & SPECTOR, A.A. (1975). Fatty acid biosynthesis in Ehrlich cells. The mechanism of short term control by exogenous free fatty acids. *J. Biol. Chem.*, **250**, 5419.

MINOURA, T., TAKATA, T., SAKAGUCHI, M. & 4 others (1988). Effect of dietary eicosapentaenoic acid on azoxymethan-induced colon carcinogenesis in rats. *Cancer Res.*, **48**, 4790.

NEOPTOLEMOS, J.P. & THOMAS, B.S. (1990). Red cell membrane fatty acid profiles in colorectal cancer using tube capillary gas chromatography. *Ann. Clin. Biochem.*, **29**, 38.

NICHOLSON, M.L., NEOPTOLEMOS, J.P., CLAYTON, H.A. & HEAGERTY, A.M. (1988). Diet and colorectal cancer. *Int. Clin. Nutr. Rev.*, **8**, 180.

NICHOLSON, M.L., NEOPTOLEMOS, J.P., CLAYTON, H.A., TALBOT, I.C. & BELL, P.R.F. (1990*a*). Increased arachidonic acid in cell membranes of experimental colorectal tumours. *Gut* (in the press).

NICHOLSON, M.L., NEOPTOLEMOS, J.P., CLAYTON, H.A., TALBOT, I.C. & BELL, P.R.F. (1990*b*). Fatty acids in experimental colorectal carcinogenesis. (Abstract). *Br. J. Surg.* (in the press).

NIGRO, N.D., SINGH, D.V., CAMPBELL, R.L. & PAK, M.S. (1975). Effect of dietary beef fat on intestinal tumour formation by azoxymethane in rats. *J. Natl Cancer Inst.*, **54**, 439.

OOKTENS, M., MONTISANO, D., LYON, I. & BAKER, N. (1986). Inhibition of fatty acid incorporation into adipose tissue triglycerides in Ehrlich ascites tumor-bearing mice. *Cancer Res.*, **46**, 633.

POPP-SNIJDERS, C., SCHOUTEN, J.A., VAN BLITTERWIJK, W.J. & VAN DER VEEN, E.A. (1986). Changes in membrane lipid composition of human erythrocytes after dietary supplementation of (n-3) polyunsaturated fatty acids. Maintenance of membrane fluidity. *Biochim. Biophys. Acta.*, **85**, 31.

PRITCHARD, G.A., JONES, D.L. & MANSEL, R.E. (1989). Lipids in breast carcinogenesis. *Br. J. Surg.*, **76**, 1069.

REDDY, B.S. & MARUYAMA, H. (1986). Effect of dietary fish oil on azoxymethane-induced colon carcinogenesis in male F344 rats. *Cancer Res.*, **46**, 3367.

REDDY, B.S. & SUGIE, S. (1988). Effect of different levels of omega-3 and omega-6 fatty acids on azoxymethane-induced colon carcinogenesis in F344 rats. *Cancer Res.*, **48**, 6642.

REDDY, B.S., TANAKA, T. & SIMI, B. (1985). Effect of different levels of dietary *trans* fat or corn oil on azoxymethane-induced colon carcinogenesis in F.344 rats. *J. Natl Cancer Inst.*, **75**, 791.

REDDY, B.S., WATANABE, K. & WEISBURGER, J.H. (1977). Effect of high-fat diet on colon carcinogenesis in F344 rats treated with 1,2-dimethylhydrazine, methylazoxymethanol acetate or methylnitrosourea. *Cancer Res.*, **37**, 4156.

SAKAGUCHI, M., HIRAMATSU, Y., TAKADA, H. & 4 others (1984). Effect of dietary unsaturated and saturated fats on azoxymethane-induced colon carcinogenesis in rats. *Cancer Res.*, **44**, 1472.

SCHLAGER, S.E. & OHANIAN, S.H. (1980). Modulation of tumour cell susceptibility to humoral immune killing through chemical and physical manipulation of cellular lipid and fatty acid compositon. *J. Immunol.*, **125**, 1196.

SMEDLEY-MACHLEAN, I. & NUNN, L.C.A. (1941). Fat-deficiency disease of rats. The relation of the essential unsaturated acids to tumour formation of the albino rat on normal diet. *Biochem. J.*, **35**, 983.

WALD, N.S., BOREHAM, J. & BAILEY, A. (1986). Serum retinol and the subsequent risk of cancer. *Br. J. Cancer*, **54**, 957.

WICKREMASINGHE, R.G. (1988). The role of prostaglandins in the regulation of cell proliferation. *Prostaglandins Leukotrienes Essent. Fatty Acids*, **31**, 171.

WILSON, J.D. & LINDSEY, C.A. (1965). Studies on the influence of dietary cholesterol on cholesterol metabolites in the iso-topic steady state in man. *J. Clin. Invest.*, **44**, 1805.


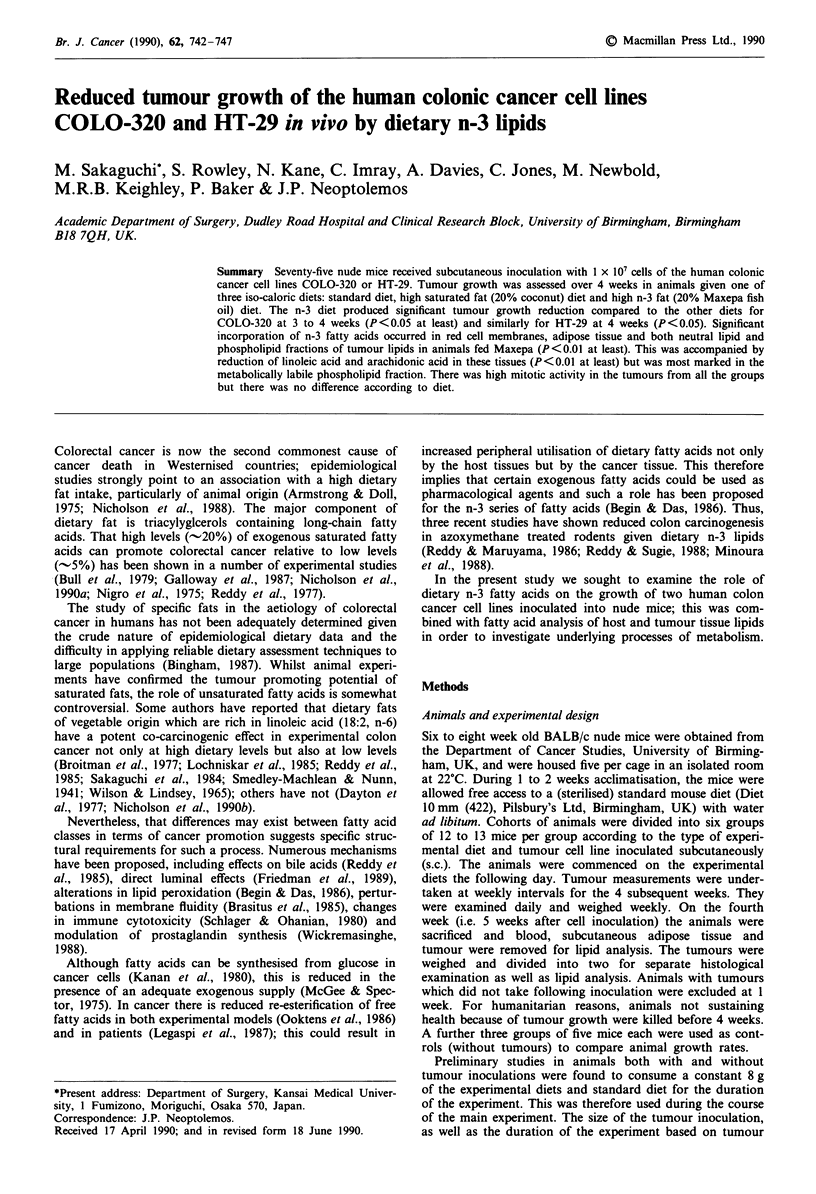

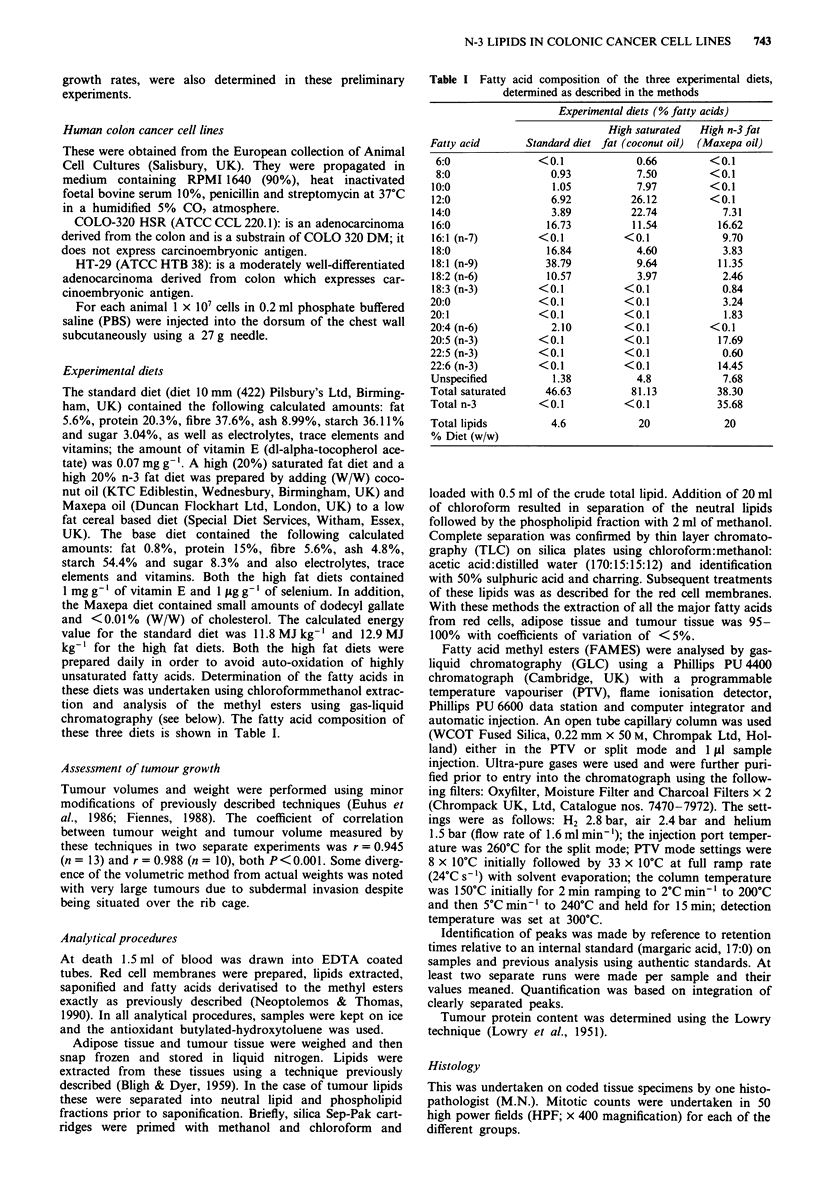

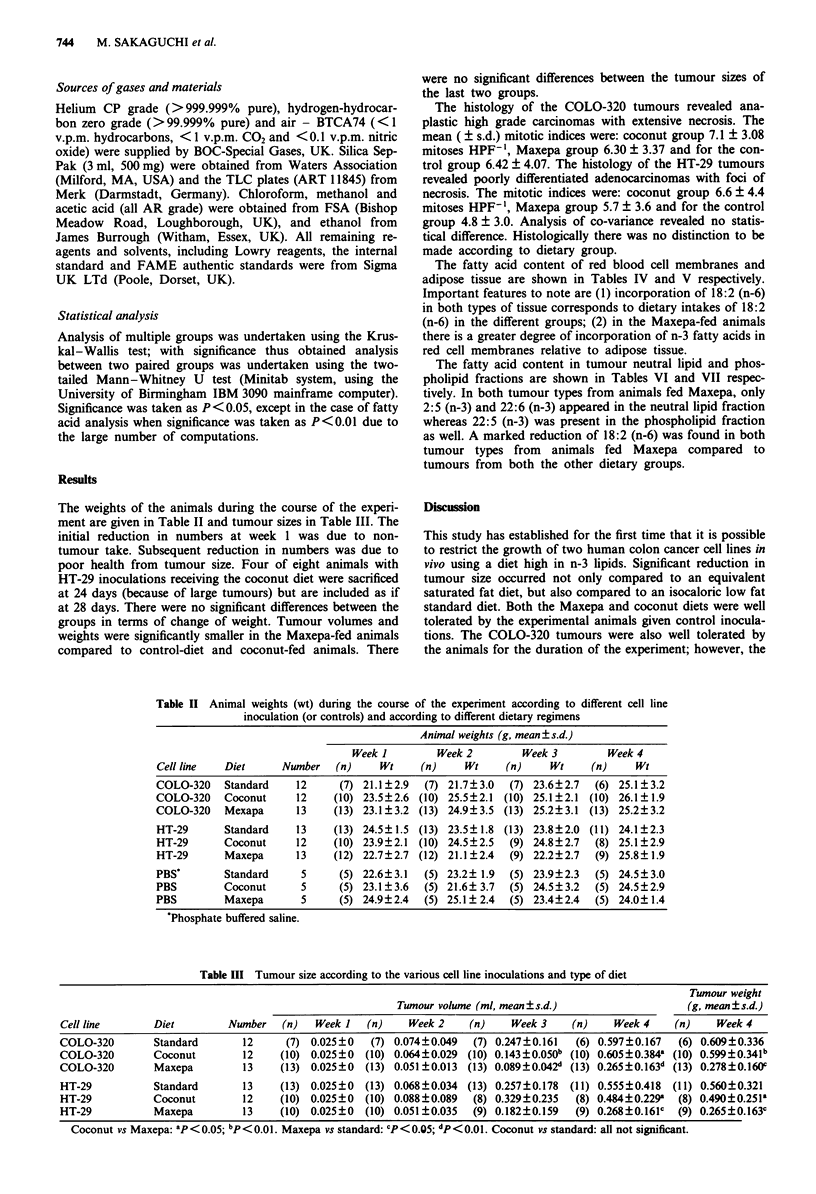

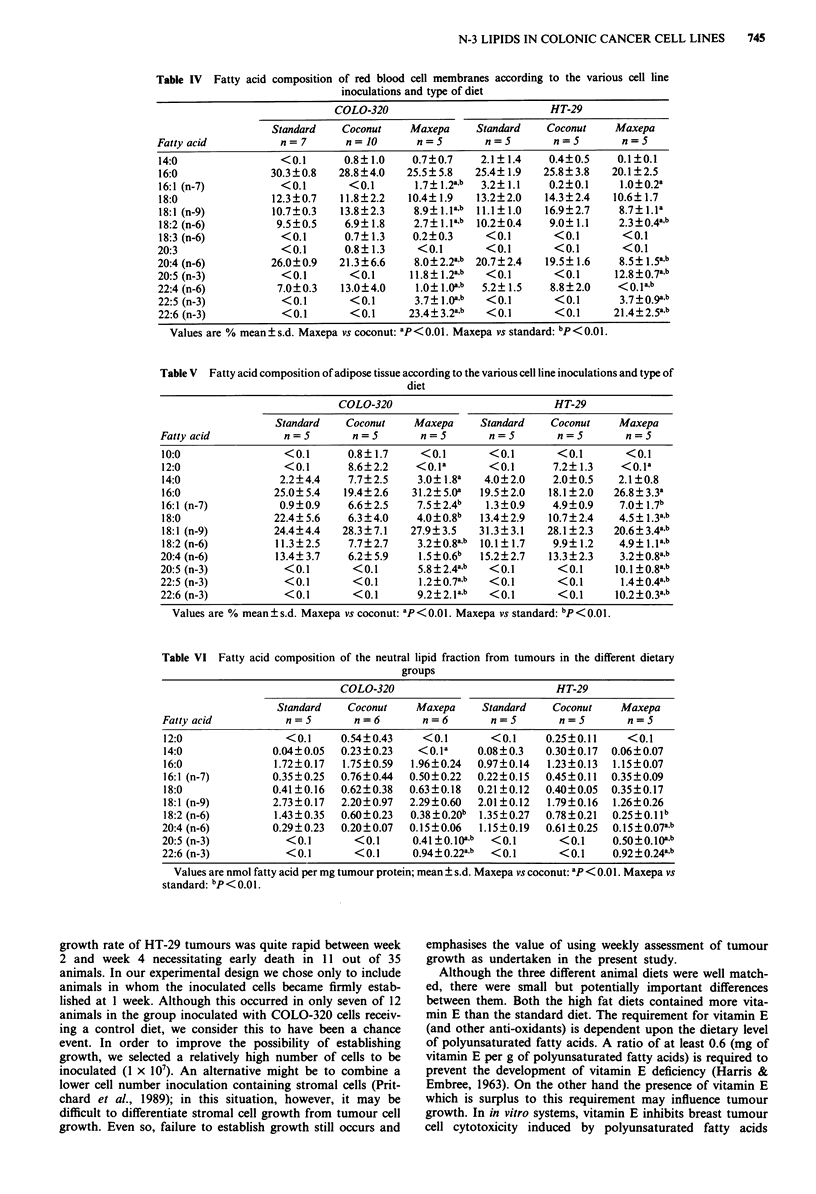

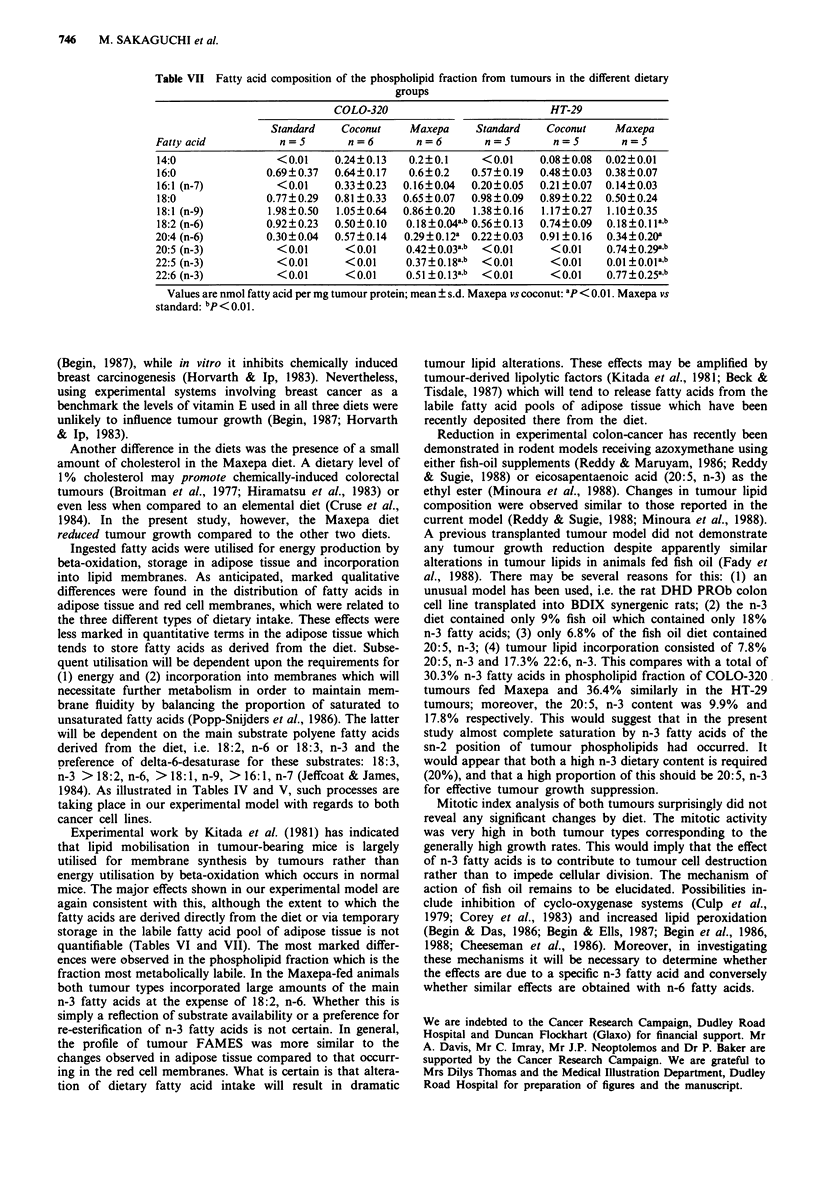

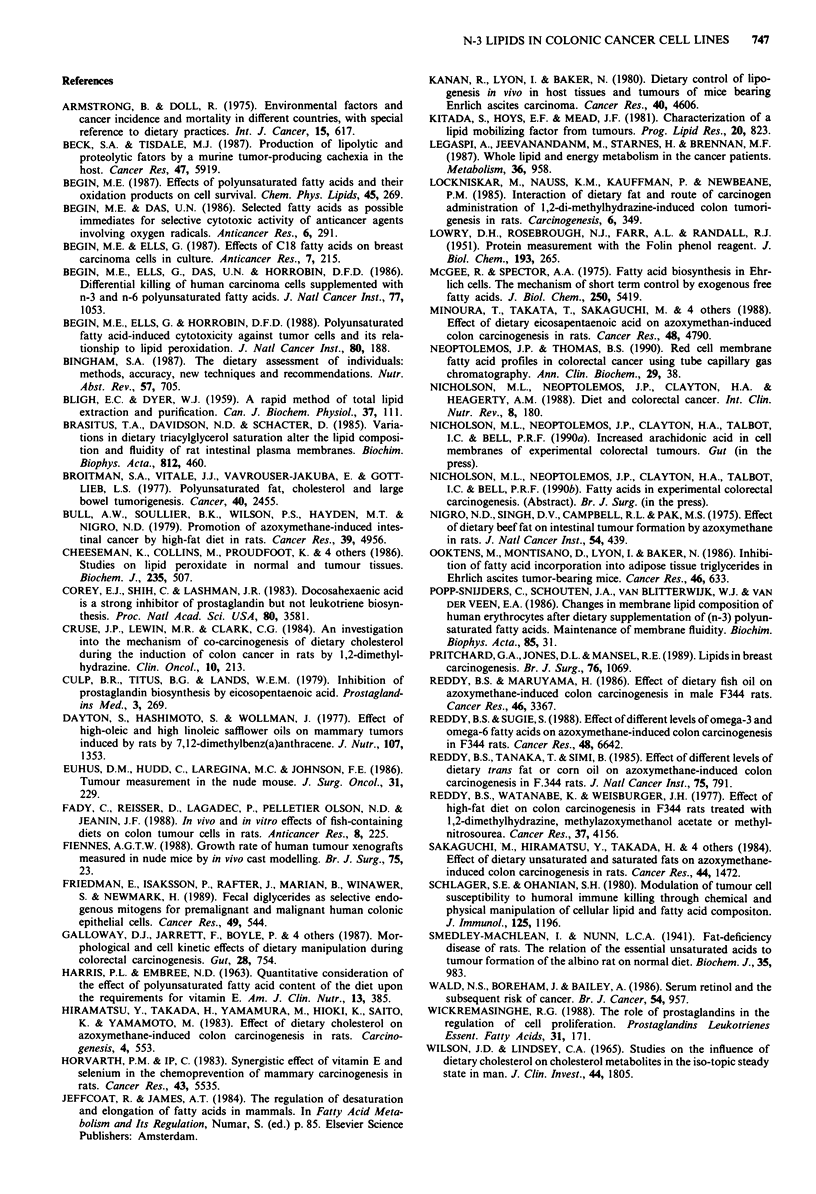


## References

[OCR_00697] Armstrong B., Doll R. (1975). Environmental factors and cancer incidence and mortality in different countries, with special reference to dietary practices.. Int J Cancer.

[OCR_00702] Beck S. A., Tisdale M. J. (1987). Production of lipolytic and proteolytic factors by a murine tumor-producing cachexia in the host.. Cancer Res.

[OCR_00738] Brasitus T. A., Davidson N. O., Schachter D. (1985). Variations in dietary triacylglycerol saturation alter the lipid composition and fluidity of rat intestinal plasma membranes.. Biochim Biophys Acta.

[OCR_00746] Broitman S. A., Vitale J. J., Vavrousek-Jakuba E., Gottlieb L. S. (1977). Polyunsaturated fat, cholesterol and large bowel tumorigenesis.. Cancer.

[OCR_00749] Bull A. W., Soullier B. K., Wilson P. S., Hayden M. T., Nigro N. D. (1979). Promotion of azoxymethane-induced intestinal cancer by high-fat diet in rats.. Cancer Res.

[OCR_00707] Bégin M. E. (1987). Effects of polyunsaturated fatty acids and of their oxidation products on cell survival.. Chem Phys Lipids.

[OCR_00719] Bégin M. E., Ells G., Das U. N., Horrobin D. F. (1986). Differential killing of human carcinoma cells supplemented with n-3 and n-6 polyunsaturated fatty acids.. J Natl Cancer Inst.

[OCR_00710] Bégin M. E., Ells G., Das U. N. (1986). Selected fatty acids as possible intermediates for selective cytotoxic activity of anticancer agents involving oxygen radicals.. Anticancer Res.

[OCR_00715] Bégin M. E., Ells G. (1987). Effects of C18 fatty acids on breast carcinoma cells in culture.. Anticancer Res.

[OCR_00725] Bégin M. E., Ells G., Horrobin D. F. (1988). Polyunsaturated fatty acid-induced cytotoxicity against tumor cells and its relationship to lipid peroxidation.. J Natl Cancer Inst.

[OCR_00754] Cheeseman K. H., Collins M., Proudfoot K., Slater T. F., Burton G. W., Webb A. C., Ingold K. U. (1986). Studies on lipid peroxidation in normal and tumour tissues. The Novikoff rat liver tumour.. Biochem J.

[OCR_00759] Corey E. J., Shih C., Cashman J. R. (1983). Docosahexaenoic acid is a strong inhibitor of prostaglandin but not leukotriene biosynthesis.. Proc Natl Acad Sci U S A.

[OCR_00764] Cruse J. P., Lewin M. R., Clark C. G. (1984). An investigation into the mechanism of co-carcinogenesis of dietary cholesterol during the induction of colon cancer in rats by 1,2 dimethylhydrazine.. Clin Oncol.

[OCR_00770] Culp B. R., Titus B. G., Lands W. E. (1979). Inhibition of prostaglandin biosynthesis by eicosapentaenoic acid.. Prostaglandins Med.

[OCR_00775] Dayton S., Hashimoto S., Wollman J. (1977). Effect of high-oleic and high-linoleic safflower oils on mammary tumors induced in rats by 7,12-dimethylbenz(alpha)anthracene.. J Nutr.

[OCR_00781] Euhus D. M., Hudd C., LaRegina M. C., Johnson F. E. (1986). Tumor measurement in the nude mouse.. J Surg Oncol.

[OCR_00788] Fady C., Reisser D., Lagadec P., Pelletier H., Olsson N. O., Jeannin J. F. (1988). In vivo and in vitro effects of fish-containing diets on colon tumour cells in rats.. Anticancer Res.

[OCR_00791] Fiennes A. G. (1988). Growth rate of human tumour xenografts measured in nude mice by in vivo cast modelling.. Br J Surg.

[OCR_00796] Friedman E., Isaksson P., Rafter J., Marian B., Winawer S., Newmark H. (1989). Fecal diglycerides as selective endogenous mitogens for premalignant and malignant human colonic epithelial cells.. Cancer Res.

[OCR_00802] Galloway D. J., Jarrett F., Boyle P., Indran M., Carr K., Owen R. W., George W. D. (1987). Morphological and cell kinetic effects of dietary manipulation during colorectal carcinogenesis.. Gut.

[OCR_00807] HARRIS P. L., EMBREE N. D. (1963). QUANTITATIVE CONSIDERATION OF THE EFFECT OF POLYUNSATURATED FATTY ACID CONTENT OF THE DIET UPON THE REQUIREMENTS FOR VITAMIN E.. Am J Clin Nutr.

[OCR_00812] Hiramatsu Y., Takada H., Yamamura M., Hioki K., Saito K., Yamamoto M. (1983). Effect of dietary cholesterol on azoxymethane-induced colon carcinogenesis in rats.. Carcinogenesis.

[OCR_00829] Kannan R., Lyon I., Baker N. (1980). Dietary control of lipogenesis in vivo in host tissues and tumors of mice bearing Ehrlich ascites carcinoma.. Cancer Res.

[OCR_00834] Kitada S., Hays E. F., Mead J. F. (1981). Characterization of a lipid mobilizing factor from tumors.. Prog Lipid Res.

[OCR_00848] LOWRY O. H., ROSEBROUGH N. J., FARR A. L., RANDALL R. J. (1951). Protein measurement with the Folin phenol reagent.. J Biol Chem.

[OCR_00837] Legaspi A., Jeevanandam M., Starnes H. F., Brennan M. F. (1987). Whole body lipid and energy metabolism in the cancer patient.. Metabolism.

[OCR_00842] Locniskar M., Nauss K. M., Kaufmann P., Newberne P. M. (1985). Interaction of dietary fat and route of carcinogen administration on 1,2-dimethylhydrazine-induced colon tumorigenesis in rats.. Carcinogenesis.

[OCR_00853] McGee R., Spector A. A. (1975). Fatty acid biosynthesis in Erlich cells. The mechanism of short term control by exogenous free fatty acids.. J Biol Chem.

[OCR_00858] Minoura T., Takata T., Sakaguchi M., Takada H., Yamamura M., Hioki K., Yamamoto M. (1988). Effect of dietary eicosapentaenoic acid on azoxymethane-induced colon carcinogenesis in rats.. Cancer Res.

[OCR_00863] Neoptolemos J. P., Thomas B. S. (1990). Erythrocyte membrane stearic acid: oleic acid ratios in colorectal cancer using tube capillary column gas liquid chromatography.. Ann Clin Biochem.

[OCR_00884] Nigro N. D., Singh D. V., Campbell R. L., Sook M. (1975). Effect of dietary beef fat on intestinal tumor formation by azoxymethane in rats.. J Natl Cancer Inst.

[OCR_00889] Ookhtens M., Montisano D., Lyon I., Baker N. (1986). Inhibition of fatty acid incorporation into adipose tissue triglycerides in Ehrlich ascites tumor-bearing mice.. Cancer Res.

[OCR_00894] Popp-Snijders C., Schouten J. A., van Blitterswijk W. J., van der Veen E. A. (1986). Changes in membrane lipid composition of human erythrocytes after dietary supplementation of (n-3) polyunsaturated fatty acids. Maintenance of membrane fluidity.. Biochim Biophys Acta.

[OCR_00901] Pritchard G. A., Jones D. L., Mansel R. E. (1989). Lipids in breast carcinogenesis.. Br J Surg.

[OCR_00905] Reddy B. S., Maruyama H. (1986). Effect of dietary fish oil on azoxymethane-induced colon carcinogenesis in male F344 rats.. Cancer Res.

[OCR_00910] Reddy B. S., Sugie S. (1988). Effect of different levels of omega-3 and omega-6 fatty acids on azoxymethane-induced colon carcinogenesis in F344 rats.. Cancer Res.

[OCR_00915] Reddy B. S., Tanaka T., Simi B. (1985). Effect of different levels of dietary trans fat or corn oil on azoxymethane-induced colon carcinogenesis in F344 rats.. J Natl Cancer Inst.

[OCR_00920] Reddy B. S., Watanabe K., Weisburger J. H. (1977). Effect of high-fat diet on colon carcinogenesis in F344 rats treated with 1,2-dimethylhydrazine, methylazoxymethanol acetate, or methylnitrosourea.. Cancer Res.

[OCR_00926] Sakaguchi M., Hiramatsu Y., Takada H., Yamamura M., Hioki K., Saito K., Yamamoto M. (1984). Effect of dietary unsaturated and saturated fats on azoxymethane-induced colon carcinogenesis in rats.. Cancer Res.

[OCR_00931] Schlager S. I., Ohanian S. H. (1980). Modulation of tumor cell susceptibility to humoral immune killing through chemical and physical manipulation of cellular lipid and fatty acid composition.. J Immunol.

[OCR_00937] Smedley-Maclean I., Nunn L. C. (1941). Fat-deficiency disease of rats. The relation of the essential unsaturated acids to tumour formation in the albino rat on normal diet.. Biochem J.

[OCR_00943] Wald N., Boreham J., Bailey A. (1986). Serum retinol and subsequent risk of cancer.. Br J Cancer.

[OCR_00947] Wickremasinghe R. G. (1988). The role of prostaglandins in the regulation of cell proliferation.. Prostaglandins Leukot Essent Fatty Acids.

[OCR_00952] Wilson J. D., Lindsey C. A. (1965). Studies on the influence of dietary cholesterol on cholesterol metabolism in the isotopic steady state in man.. J Clin Invest.

